# Preparation, Evaluation and Bioavailability Studies of Eudragit Coated PLGA Nanoparticles for Sustained Release of Eluxadoline for the Treatment of Irritable Bowel Syndrome

**DOI:** 10.3389/fphar.2017.00844

**Published:** 2017-11-20

**Authors:** Md. K. Anwer, Ramadan Al-Shdefat, Essam Ezzeldin, Saad M. Alshahrani, Abdullah S. Alshetaili, Muzaffar Iqbal

**Affiliations:** ^1^Department of Pharmaceutics, College of Pharmacy, Prince Sattam Bin Abdulaziz University, Al-Kharj, Saudi Arabia; ^2^Department of Pharmaceutical Sciences, Faculty of Pharmacy, Jadara University, Irbid, Jordan; ^3^Department of Pharmaceutical Chemistry, College of Pharmacy, King Saud University, Riyadh, Saudi Arabia; ^4^Bioavailability Laboratory, College of Pharmacy, King Saud University, Riyadh, Saudi Arabia

**Keywords:** eluxadoline, PLGA, nanoparticles, enteric coating, pharmacokinetics

## Abstract

Eluxadoline is a newly approved orally administered drug used for the treatment of Irritable Bowel Syndrome with Diarrhea. It is reported as a poorly water-soluble drug due to which its dissolution rate and oral bioavailability are very poor. In this work, various plain PLGA nanoparticles (NPs) (F1–F4) were prepared and optimized based on particle size, PDI, zeta potential and percent drug entrapment efficiency (EE). The developed plain NPs (F1–F4) showed average particle size ranging from 260.19 to 279.76 nm with smooth surface and EE of 17.83–56.29%. The optimized plain NPs (F3) had particle size of 273.76 ± 7.25 nm with a low PDI value 0.327, zeta potential - 30.63 ± 2.47 mV and % EE of 56.29 ± 2.56%. The optimized F3 NPs was further submitted for enteric coating using Eudragit S100 polymer and evaluated in terms of particles characterization, *in vitro* release and pharmacokinetic studies in rats. The bioavailability of plain and coated nanaoparticles were enhanced by 6.8- and 18.5-fold, respectively, compared to normal suspension. These results revealed that the developed coated NPs could be used for its oral delivery for an effective treatment of Irritable Bowel Syndrome with Diarrhea.

## Introduction

Irritable Bowel Syndrome with Diarrhea (IBS-D) is a functional gastrointestinal disorder in adults with symptoms of that include abdominal pain or discomfort, sudden urges to have bowel movements, gas, and frequent stools. In many cases of IBS, the underlying cause remains unknown and therefore its treatment are based on improving the symptoms of the disease (Camilleri et al., 2002; Talley et al., 2003).

Eluxadoline (ELUX) is a recently approved an orallay active drug, indicated for the treatment of IBS-D by targeting mixed μ-and κ-opioid receptor agonist, and δ opioid receptor antagonist in the gastrointestinal tract which lead to improve IBS-D symptoms and help to regulate colon function (Davenport et al., 2015; Lacy and Moreau, 2016). ELUX has a very low oral bioavailability (1%) with high inter-subject variability (51–98%) in humans. Poor bioavailability is mainly due to limited absorption and first pass effects (Lamprecht et al., 2001; Dai et al., 2004; Ali et al., 2014; Lembo et al., 2016). The peak plasma concentration (*C*_max_) and area under curve (AUC) was found to approximately 2–4 ng/mL and 12–22 ng.h/mL, respectively, in healthy volunteers after single oral dose administration of 100 mg tablets (Pharmaceutical Industries Ltd., 2015; Allergan Ltd., 2017). In addition, ELUX has been reported as a poorly water-soluble drug due to which its dissolution rate is poor which in turns may results in poor oral bioavailability (Allergan Pharma Co., 2017). It is commercially available in tablet dosage form with strength of 75 and 100 mg for the treatment of IBS-D. Capsules and Liquid formulation of ELUX for pediatric use is not available commercially (Allergan Pharma Co., 2017). Due to poor bioavailability and high inter-subject variability of conventional formulation, a novel sustained release formulation of ELUX is prerequisite for its efficient delivery intended to IBS-D treatment.

Drug delivery to the target site of action is one of the main challenges for effective treatment and subsiding the adverse effects of the drugs. This obstacle might happen due to various factors, e.g., physicochemical properties of drugs, ADME profiles or patient factors (age, disease etc.) (Perrie et al., 2012). Nanotechnology is a novel approach for drug delivery which overcome above limitations and provide benefits in term of bioavailability, solubility, selective targeting and control release of drug formulations (Luo et al., 2011; Danhier et al., 2012). Recently, Wei et al. (2016) enhanced the oral bioavailability of loperamide by solid lipid nanoparticle approach and concluded that lipid based nanoparticles provides a effective tool to enhance the oral bioavailability of poorly water soluble drugs. The polymer based nanoparticulate drug delivery systems have also been known to be an efficient approach to enhance drug absorption, improve bioavailability, targeting of therapeutic agents to colon and reduce toxicity (Anwer et al., 2016a,b). However, these colon-specific delivery systems have some limitations due to large variations in gastric emptying time and in pH of the gastrointestinal tract in addition to initial burst release (Yassin et al., 2010; Anwer et al., 2016a,b).

In this work, we investigated the ability of Eudragit S100 coated PLGA based pH sensitive nanoparticles to entrap and release ELUX in a controlled manner for colon targeted-drug delivery in IBS-D. We synthesized core-shell nanoparticles in which PLGA is being core and Eudragit S100 being an external shell on nanoparticles surface. Single-emulsion solvent evaporation technique was used to synthesize Eudragit coated ELUX loaded pH- sensitive nanoparticles. For comparative study, ELUX loaded PLGA nanoparticles (PLGA NPs) without coating were also prepared by the same technique. The physiochemical and surface properties of the different formulations were studied, also *in vitro* drug release profiles were evaluated at different pH. Finally, the pharmacokinetics and bioavailability of optimized formulations were compared with normal suspension.

## Materials and Methods

### Materials

ELUX was purchased from Beijing Mesochem Technology Co., Ltd., China (PLGA)Poly(D,L-lactide-co-glycolide) (lactide:glycolide ratio of 75:25) were purchased from Lactel Absorbable Polymer (Pelham, AL, United States). Eudragit S100 and Poly(vinyl) alcohol were purchased from Sigma–Aldrich (St. Louis, MO, United States). All reagents/chemicals used in the experiment were analytical grade.

### Preparation of ELUX Loaded PLGA and Coated PLGA Nanoparticles

PLGA polymeric nanoparticles were developed by single emulsion and evaporation technique (Ali et al., 2014). Briefly, ELUX (10 mg) was solubilized in 5 ml of ethyl acetate containing PLGA polymer (50–200 mg). This organic phase was further emulsified drop wise to an aqueous solution containing PVA (0.5% w/v) with the help of stirring for 6 h at room temperature. The organic solvent, ethyl acetate was evaporated under reduced pressure at 40°C. The ELUX loaded PLGA NPs were collected from bulk aqueous phase by centrifugation at 20000 rpm for 25 min followed by subsequent three times washing with cold deionized water and lyophilized. The PLGA NPs were further coated with enteric coating polymer (Eudragit S100). Eudragit S100 (5% w/v) polymer was dissolved in a mixture of 5 mL 0.5% PVA, 10 ml methanol and 5 ml 0.1 N NaOH. The obtained plain PLGA NPs were further added drop wise to above prepared enteric coating solution on magnetic stirrer. The developed emulsion was further sonicated by probe sonication at 40% W for 3 min.

### Particle Characteristics

The average particle size, PDI and zeta potential of developed ELUX loaded plain PLGA NPs (F1–F4) and its coated nanoparticles (F3C) were determined using “Malvern Particle Size Analyzer (Malvern Instruments Ltd., Holtsville, NY, United States)” at 25 ± 1°C. The scattering angle 90° was fixed for the measurement. The suspension of plain and coated NPs were diluted 1:100 times with double distilled water to obtain uniform dispersion. Each samples were sonicated for 5 min and transferred to plastic cuvettes and analyzed in triplicate for average particle size and PDI. The values of zeta potential of each sample (F1–F4) were obtained using “Malvern Zetasizer.” The samples were transferred to glass electrodes for ZP measurement (Mainardes and Evangelista, 2005; Ramani et al., 2011).

### Percent Entrapment Efficiency

To determine the percent entrapment efficiency (EE), a known quantity of ELUX loaded plain and coated PLGA NPs were first dissolved in deionized water. The nanosuspension was then separated from the unentrapped drug using membrane filter and the amount of free drug in the filtrate was measured using HPLC technique (Prasad and Kumar, 2015). The percent EE were determined as the ratio of the amount of drug incorporated in nano-particles to the amount used for the preparation of nano-particles. The experiments were conducted in triplicate.

$$\%EE\;=\;[(Drug\;added-Free\text{ '}unentrapped\;drug\text{'})\text{/Drug added}]{\;}^{\text{*}}\;\text{100}$$

### *In Vitro* Drug Release Profile

Release profile of ELUX pure, plain and coated PLGA NPs were tested in progressive pHs, simulated gastric fluid (SGF, pH-1.2) and simulated intestinal fluid (SIF, pH-7.4). Initially, pH 1.2 media was used for 2 h of the study and further dissolution was performed at pH 7.4 media till 24 h. Accurately weighed 2.5 mg of dry powder of ELUX pure, plain and coated NPs were dispersed in 5 mL media and placed in treated dialysis bag (cut off # 12000) in 35 mL in media. The dialysis bags were incubated at 37 ± 0.5°C with shaking at 100 rpm in a biological shaker (LBS-030S-Lab Tech, Korea). 1.0 mL of aliquots were withdrawn, compensated with fresh media at a different time points 0, 0.5, 1, 2, 3, 4, 5, 6, 12, and 24 h and analyzed for the drug content by HPLC method. A reported HPLC method was used after slight modification (Prasad and Kumar, 2015). The release of drug from optimized PLGA NPs (F3) and its coated NPs (F3C) were compared with the pure ELUX drug powder.

### Fourier Transform Infrared Spectroscopy (FT-IR)

The FTIR spectra of samples were recorded in a ALPHA FT-IR Spectrometer (OPTIK, United States) using the potassium bromide (KBr) disk technique. Samples equivalent to 2 mg of ELUX, plain (F3) and coated (F3C) PLGA NPs were diluted with potassium bromide (about 100 mg) in a clean glass pestle and mortar and were compressed to obtain transparent pellets (Fatma et al., 2016). The baseline was corrected and the samples were scanned against a blank KBr pellet at a wave number in the range of 4000–400 cm^-1^.

### Powder X-Ray Diffraction Pattern (XRD)

The powder X-ray diffraction (XRD) of ELUX, plain and coated PLGA NPs were recorded in an X-ray diffractometer (Altima IV Regaco, Japan). The scanning rate of the spectra was 4°/min. The voltage/current used was 30 kV/25 mA and the target/filter (monochromator) was copper (Ramani et al., 2011).

### Scanning Electron Microscopic (SEM) Analysis

The images plain (F3) and coated (F3C) PLGA NPs was recorded by a scanning electron microscope (JSM-6360LV Scanning Microscope; Jeol, Tokyo, Japan). Suspended NPs were mixed for 5 min, and then a drop was smeared on a glass slide and kept in a desiccator to dry. The dried sample was mounted on a carbon tape and sputter-coated using a thin gold palladium layer under argon using a gold sputter module in a high-vacuum evaporator (JFC-1100 fine coat ion sputter; Jeol, Tokyo, Japan). The coated samples were then scanned and photographed (Fatma et al., 2016).

### Bioanalytical Methods

The UPLC-MS/MS instrument consisting of H-class Aquity UPLC connected to tandem quadruple detector (Waters Corp., Milford, MA, United States) was used for the analysis of ELUX in plasma samples. It was operated in electrospray ionization in positive mode and multiple reaction monitoring (MRM) mode was used for the samples detection and quantification. MRM transition of 570.16 → 118.12 for qualifier and 570.16 → 171.08 for quantifier were used for ELUX whereas MRM transition of 411.18 → 191.07 was used for IS (risperidone) analysis. The MS/MS operating conditions, e.g., capillary voltage (2.4 Kv) source temperature (150°C) desolvation temperature (350°C) desolation gas flow (600 L/h) and collision (argon) gas flow (0.15 mL/ min) were carefully to optimized to achieve best ionization condition. MassLynx software (Version 4.1) was used to operate the instrument and the Target Lynx^TM^ program was used for data processing. Both ELUX and IS were separated on C_18_ column (Acquity UPLC^®^ BEH, 100 mm × 2.1 mm, 1.7 μm) maintaining oven temperature of 40°C. The mobile phase mixture in a ratio of 80:20 (v/v) acetonitrile and 20 mM ammonium acetate were eluted for chromatographic separation of analyte and IS at the flow rate of 0.3 mL/ min. Both ELUX and IS were eluted at 0.84 and 1.04 min, respectively, with the total run time of 2.0 min only.

The μ-SPE procedure was used for sample extraction from plasma. Initially the samples (100 μL) were diluted with an equivalent amount of 4% *o*-phosphoric acid and were cold centrifuged at 4500 × *g* for 5 min. Then the diluted samples were loaded to Oasis MCX 96 μ-elution well-plate (Waters, Milford, MA, United States). The plate was preconditioned and equilibrated by 200 μL of methanol and water, respectively. After washing by 200 μL of 2 % formic acid and methanol, the samples were eluted by using 100 μL of 5% ammonia dissolved in mixture of acetonitrile:methanol (60:40, v/v) and collected into a 96-well collection plate. The eluted samples were directly injected into the UPLC system for analysis.

### Pharmacokinetic Study in Rats

Eighteen male Wistar albino rats weighing 180–220 g were procured from the Animal care and use centre, College of Pharmacy, King Saud University, Riyadh, Saudi Arabia. The animal protocol to carry out *in vivo* study was reviewed and approved by Institutional Animal Ethics Committee, King Saud University, Riyadh, Saudi Arabia and their guidelines were followed for the studies. Prior to the test, the rats were kept in plastic enclosures under standard research center conditions, temperature 25 ± 2°C and %RH 55 ± 5% with a 12 h light/dim cycle and pellet diet was given with water *ad libitum*. Rats were randomly divided into three groups (*n* = 6) according to single-dose parallel study, which served as ELUX suspension (0.5% HPMC), optimized plain PLGA NPs (F3) and coated PLGA NPs (F3C) treatment groups, respectively. Blood samples (0.3 mL) were withdrawn from the retro-orbital plexus into heparinized microfuge tubes at different time point (predose, 0.2, 0.4, 1, 1.5, 2, 3, 5, 8, 12, and 24 h) after administration of ELUX (equivalent to 20 mg/kg, oral) in all three groups. Blood samples were centrifuged at 45,000 × *g* for 8 min to collect plasma and stored frozen at 80 ± 10°C till further analysis.

### Pharmacokinetic Calculation and Data Analysis

The ELUX concentration at different time intervals were calculated by using the plasma calibration curve and drug concentration–time profile were plotted. The pharmacokinetic parametres were calculated by WinNonlin software (Pharsight Co., Mountain View, CA, United States) and all values are expressed as the mean ± standard deviation (SD). The non-compartmental pharmacokinetic model was used to calculate *C*_max_ and time to reach maximum concentration (*T*_max_), AUC from 0 to t (AUC0–24) and 0-inf (AUC0–inf), elimination rate constant (kz), half-life (*T*½) and mean residence time (MRT).

### Statistical Analysis

Physicochemical parameters and *in vitro* drug release data were evaluated by one-way ANOVA using Dunnett’s test. However, one-way ANOVA and Tukey-Kramer multiple comparison test was used for statistical evaluation of pharmacokinetic parameters. GraphPad Instat software was used for statistical analysis, and *P* < 0.05 was considered as significant.

## Results

### Particle Characterization

**FIGURE 1:**
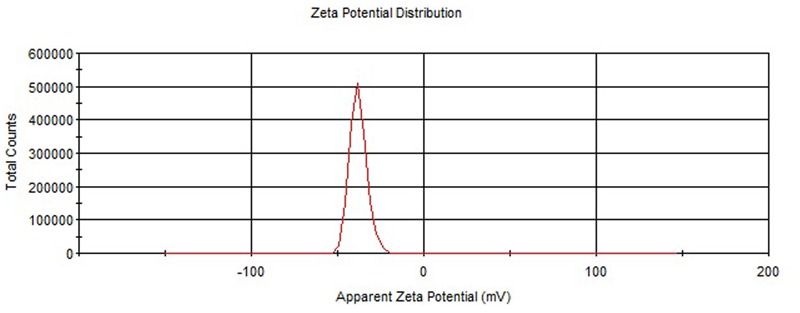
Zeta potential of plain PLGA NPs.

**FIGURE 2:**
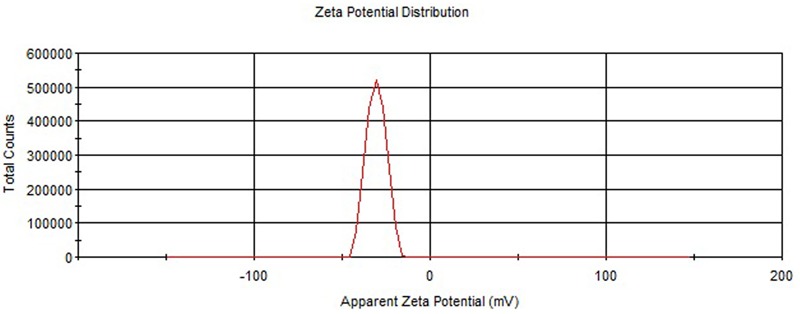
Zeta potential of Eudragit coate PLGA NPs.

The values of particle size, PDI and ZP of ELUX loaded plain PLGA NPs and coated NPs are documented in **Table 1**. The Particle size of plain/uncoated NPs (F1–F4) were measured in the range of 260.19 ± 6.34–279.76 ± 7.25 nm. The effect of the amount of PLGA polymer on size, PDI and ZP of formed plain NPs were investigated in this research. It could be observed from the data that particle size of ELUX loaded PLGA NPs increased slightly different with increase in the concentration of PLGA polymer in the formulation. The average particle size was obtained in optimized F3 (273.76 ± 7.25) with an excellent EE (56.29%). The optimized plain PLGA NPs (F3) was further selected for enteric coating with Eudragit S100 polymer. However, The size of coated PLGA NPs (F3C) was found bigger (302.06 ± 5.62) as compared optimized plain NPs. The covering of coating layer (Eudragit S100) on plain PLGA NPs increased the size of coated NPs. The PDI values of plain PLGA NPs (F1–F4) and coated were between 0.114 and 0.431, revealing its particles uniform distribution. The ZP data of plain PLGA NPs (F1–F4 and F3C) were obtained in the range of -30.63 ± 2.47 to -40.31 ± 1.56 mV. The results of ZP revealed that the all developed NPs (F1–F3) were stable because these values were higher than -30.0 mV (**Figures 1, 2**).

**Table 1:** Particle characterization of developed nanoparticles.

Code	Size (Mean ± SD)	PDI	ZP, mV (Mean ± SD)	% EE (Mean ± SD)
F1	260.32 ± 4.52	0.114	-35.23 ± 2.54	17.83 ± 1.45
F2	260.19 ± 6.34	0.107	-40.31 ± 1.56	27.45 ± 2.06
F3	273.76 ± 7.25	0.327	-30.63 ± 2.47	56.29 ± 2.56
F4	279.76 ± 7.25	0.345	-33.18 ± 4.54	44.64 ± 3.67
F3C	302.06 ± 5.62	0.431	-38.18 ± 2.07	81.23 ± 3.39

### Percent Entrapment Efficiency

The amount of PLGA polymer was found to influence the degree of ELUX entrapment inside PLGA NPs. Formulae (F3) showed maximum entrapment (56.29%) of ELUX amongst the other plain formulae (F1, F2, and F4). Increasing the amount of PLGA polymer from 50 to 150 mg (F1–F3) was found to have a positive effect on EE. However, further increase in amount of PLGA polymer above 150 mg in formulae F4 showed decrease in EE. Eudragit coated NPs (F3C) exhibited highest EE (81.23%) among all formulae probably due to coating that may minimize the leakage of drugs from NPs (**Table 1**).

### *In Vitro* Drug Release Profile

**FIGURE 3:**
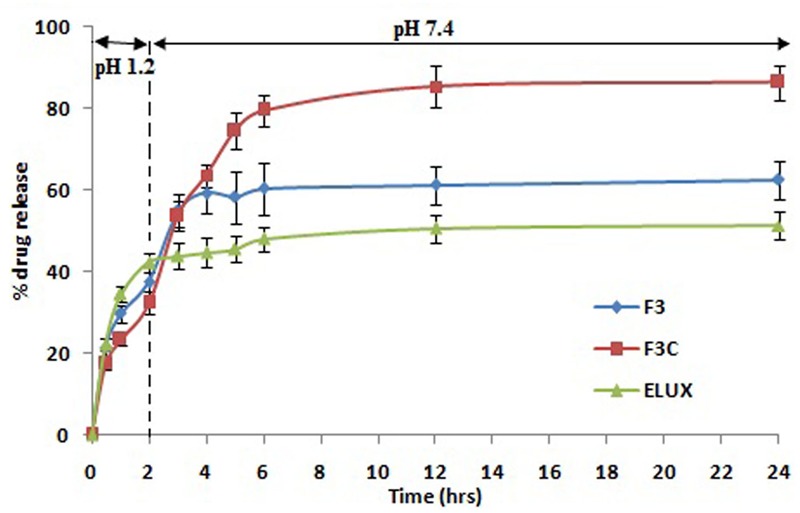
Comparative *in vitro* release profile.

*In vitro* drug release profile of pure, plain and coated PLGA NPs were tested at progressive pH 1.2 and 7.4 for 24 h (**Figure 3**). The initial burst release could be seen in pure ELUX (42.3%) after 2 h at pH 1.2, but in the case of plain NPs and coated NPs it were released less about 37.6 and 32.4%, respectively, as it was protected from acidic environment due to pH sensitive Eudragit S100 polymeric coating. However, when coated NPs (F3C) reaches in the intestine (pH 7.4), the release of ELUX was recorded maximum (86.4%) as compared to plain NPs (62.6%) and pure ELUX drug (51.3%) after 24 h. It was revealed from the results that coated NPs showed a slow release at acidic pH which reached maximum at pH 7.4 with sustained pattern of release (Ali et al., 2014).

### Fourier Transform Infrared Spectroscopy (FT-IR)

**FIGURE 4:**
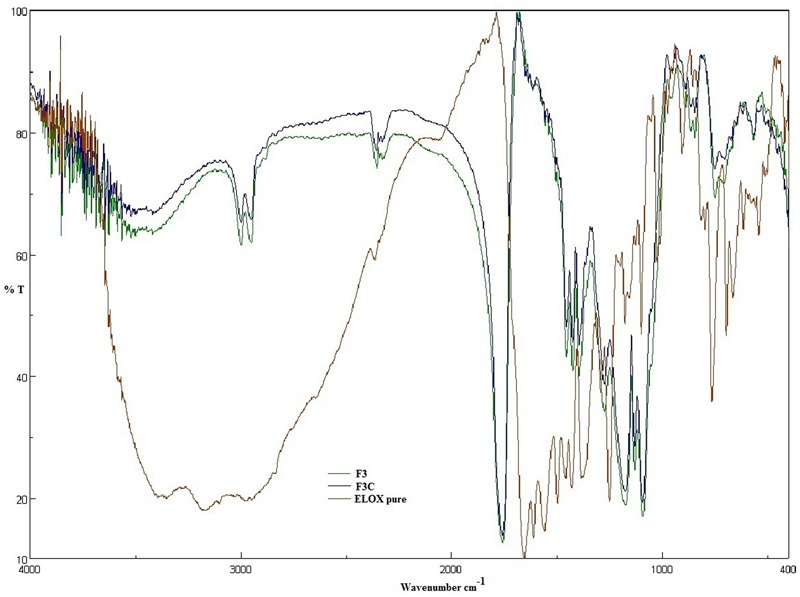
FTIR spectra of Eluxadoline (ELUX), plain and coated PLGA NPs.

The FT-IR spectra of ELUX showed a characteristic sharp peaks in the finger print region 400 cm^-1^–1600 cm^-1^. A significant change was observed in the spectra of the formulations as illustrated in **Figure 4**. All the characteristic absorption bands of ELUX diminished significantly in the finger print region of the drug in plain and coated PLGA NPs, which revealed that the encapsulated ELUX inside the PLGA polymer existed in an amorphous state.

### Powder X-Ray Diffraction Pattern (XRD)

**FIGURE 5:**
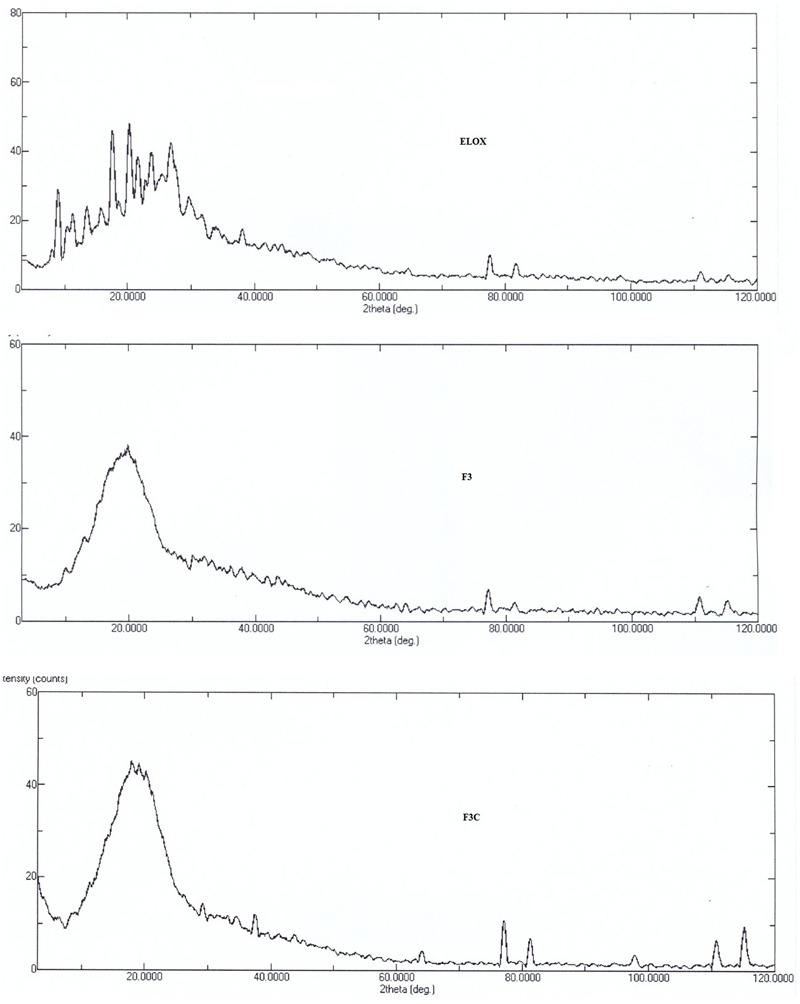
XRF spectra of ELUX, plain and coated PLGA NPs.

The powder X-ray diffraction studies are important tool to identify the nature of powder, i.e., crystal or amorphous. The XRD diffraction patterns of free ELUX, F3 and F3C NPs presented in **Figure 5**, revealed several sharp peaks could be seen in pure ELUX; however, these peaks disappeared or reduced in intensity in the ELUX loaded PLGA NPs, an indication that ELUX was encapsulated in PLGA polymer.

### Scanning Electron Microscopic (SEM) Analysis

**FIGURE 6:**
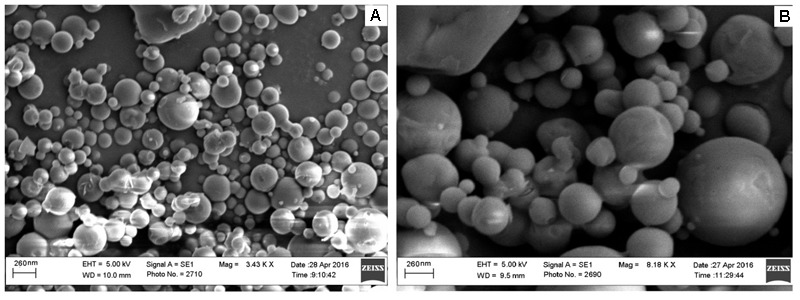
Scanning electron microscopic (SEM) images of **(A)** plain and **(B)** coated PLGA NPs.

The SEM images of ELUX loaded PLGA NPs (F3) and coated NPs are presented in **Figure 6**, It could be seen that the both plain and coated NPs were spherical in shape and have smooth surface with some agglomeration. Coated NPs were slightly bigger in size as compared to plain NPs, it may be due to surface coating of plain nanoparticles with Eudragit S100 polymer. The particle size observed from SEM image strongly supports the result of particle size analyzer.

### Pharmacokinetic Studies in Rats

**FIGURE 7:**
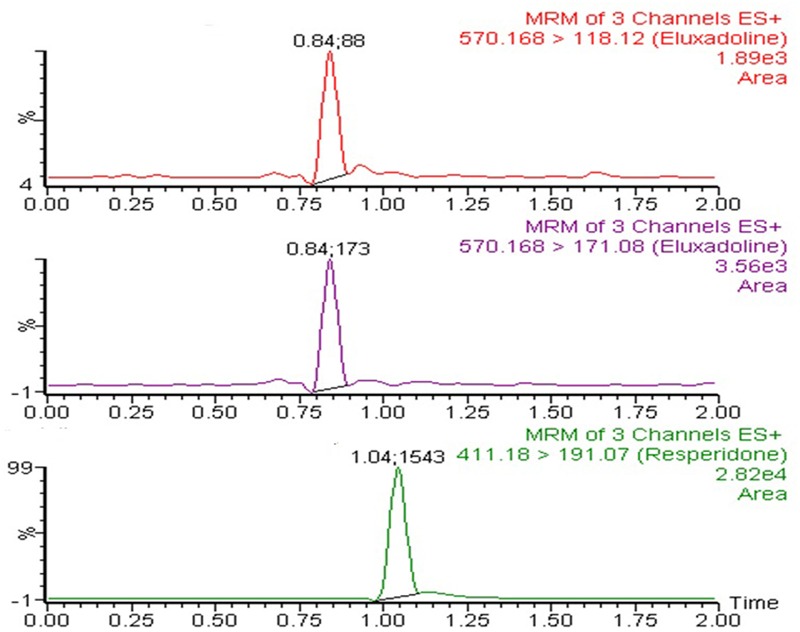
Multiple reaction monitoring (MRM) chromatogram of ELUX and IS in plasma samples after administration of ELUX normal suspension.

**FIGURE 8:**
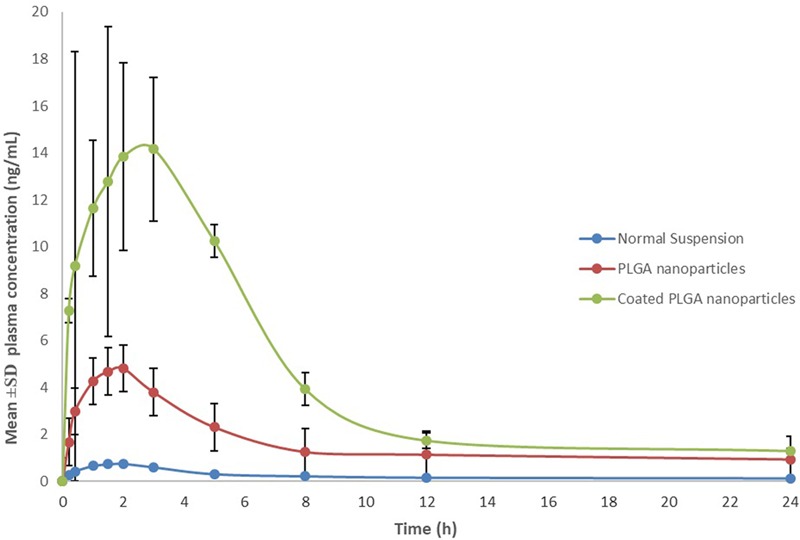
Mean ± SD plasma concentration vs. time profiles of ELUX.

Based on characterization and *in vitro* release studies, the optimized formulation F3 (Plain NPs) and F3C (Eudragit coated NPs) were submitted for the bioavailability and pharmacokinetic studies. The developed UPLC-MS/MS method was employed in the comparative bioavailability and pharmacokinetic studies of the different formulation of ELUX. The values of pharmacokinetic data of F3, F3C and normal drug suspension are documented in **Table 2**. As evident in **Table 2** the peak plasma concentrations of 0.77, 5.09 and 17.389 ng/ml were achieved at 1.75, 2, and 1.5 h for normal drug suspension, F3 and F3C, respectively. The difference in *C*_max_ of F3 and F3C was found to be highly (*P* < 0.01) and extremely (*P* < 0.001) significant, respectively, as compared to ELUX suspension. AUC0-last of normal drug suspension, F3 and F3C was recorded as 5.96, 40.10, and 110.57 ng.h/ml, respectively, whereas AUC_0-inf_ were recorded as 7.30, 54.57, and 120.05 ng.h/ml, respectively, and both were extremely significant (*P* < 0.001) as compared to ELUX suspension. Based on above results, the relative bioavailability of F3 and F3Cwere found to be increase to 6.8- and 18.5-fold, respectively, compared to normal suspension. As compared to above results there is no significant difference between *T*_1/2_ (h) normal drug suspension, F3 and F3C formulation, however, there is significant difference (*P* < 0.01) in the *K_el_*(h) value of F3C formulation and normal suspension. There is significant difference in MRT value of F3 (*P* < 0.05) and F3C (*P* < 0.01) formulation as compared to normal suspension. The representative MRM chromatogram of ELUX and IS in plasma samples after administration of ELUX normal suspension is shown in **Figure 7**, whereas the mean ± SD plasma concentration vs. time profiles ELUX after administration of 20 mg/kg of normal suspension, F3 and F3C formulation are shown **Figure 8**.

**Table 2:** Pharmacokinetic profile of developed nanoparticles.

Parameters	Standard	F3	F3C
	(Mean ±*SD*)	(Mean ±*SD*)	(Mean ±*SD*)
*C*_max_ (ng/ml)	0.77 ± 0.02	5.09 ± 0.37^∗∗^	17.39 ± 3.48 ^∗∗∗^
*T*_max_ (h)	1.75	2	1.5
AUC_last_ (ng.h/ml)	5.96 ± 0.58	40.50 ± 0.82^∗∗∗^	110.57 ± 8.36^∗∗∗^
AUC_tot_ (ng.h/ml)	7.30 ± 1.00	54.57 ± 4.45^∗∗∗^	120.05 ± 10.32^∗∗∗^
*K*_el_ (h)	0.07 ± 0.01	0.05 ± 0.01	0.11 ± 0.03^∗∗^
*T*_1/2_ (h)	9.34 ± 1.35	13.06 ± 3.81	6.66 ± 1.74
MRT (h)	13.54 ± 2.18	17.37 ± 2.98^∗^	8.46 ± 1.54^∗∗^
Relative Bioavailability (%)	100	679.25	1854.27

*^∗^*P* < 0.05 significant compared with standard suspension; ^∗∗^*P* < 0.01 highly significant compared with normal suspension and ^∗∗∗^*P* < 0.001 extremely significant compared with normal suspension.*

## Discussion

The main objective of this work was to enhance solubility and bioavailability of ELUX and sustained the release in intestinal region for the effective treatment of IBS-D. Previously we have successfully improved the bioavailability of poorly bioavailable drug, ibrutinib (absolute bioavailability 2.9%) by self-nanoemulsifying drug delivery system (Janssen Pharmaceutica NV, 2016; Shakeel et al., 2016). The alternative therapy available for IBS-D were limited due to its adverse effects mainly due to non-targeting of drugs. pH sensitive polymeric coated NPs exhibits new potential outcomes to overcome these limitations. It is possible to deliver the drug entrapped NPs inside the target site (Ali, 2013). Herein, it was expected to develop a PLGA polymeric NPs which was further enteric coated with pH sensitive polymer (Eudragit S100) with the intention of reaching these NPs more stable when it come in contact with physiological fluids. The coating with Eudragit S100 may protect the drug ELUX from harsh environment of gastric fluid.

As per authors knowledge, not a single literature are available reporting polymeric NPs of ELUX. Recent studies have shown the use of pH sensitive polymer based nanoparticles in the reformulation of budesonide, curcumin for the treatment of inflammatory bowel disease (Ali et al., 2014; Beloqui et al., 2014).

In the current study, plain PLGA NPs (F1–F4) were prepared and optimized based on preliminary characterization (**Table 1**). The formulae (F3) was found optimal and selected for further enteric coating with pH sensitive Eudragit S100 polymer. The coated PLGA NPs (F3C) was showed maximum EE among all the formulae as coating with pH sensitive polymer minimizes the leakage of drug from coated NPs. The Eudragit coated NPs (F3C) exhibited maximum entrapment (81.23%) as compared to plain PLGA NPs, which might be due to surface drug present on plain PLGA NPs was further coated with Eudragit PLGA polymer. *In vitro* release studies, a burst/fast release pattern could be seen till 2 h of the study in acidic environment in the case of pure ELUX drug, however, plain and coated NPs reduced the burst/fast release of drug (Ali et al., 2014). After 2 h drug release were sustained in all formulae (**Figure 3**). The sustained release of the drug is caused by the degradation of drug polymeric matrix. The results generated from the *in vitro* release study supported our objective that coated NPs reduce the burst release at acidic pH as compared to plain PLGA NPs. However, initial burst release of drug from coated PLGA NPs (32.4%) might be due to some non-entrap drug on the surface of nanoparticles. The Eudragit S100 polymer minimizes the burst release at acidic pH, that results in effective release of drug at higher pH. Sudden release of medication in the body can quickly achieve an effective therapeutic concentration, and sustained release can make the drug in the body to stay at the effective concentration for longer period of time (Fan et al., 2013). However, release profile of coated PLGA NPs exhibited significant and sustained drug release pattern for 24 h of the study as compared to plain PLGA NPs and ELUX pure drug (*P* < 0.05). It could be due to loss of minimum drug from coated NPs as it was protected with pH sensitive polymer Eudragit S100 and release drug maximum at pH 7.4.

FTIR spectra of plain and coated PLGA NPs showed peaks with reduced in intensity as compared to pure ELUX drug, the result revealed that maximum drug was encapsulated inside the polymer. Pure ELUX exhibited characteristic signals of their crystalline structures. The ELUX loaded plain and Eudragit coated PLGA NPs exhibited a number of different peaks; however, these peaks were slightly shifted from original positions, reduced in intensity, and also some disappeared. This indicated that the crystal form of drug was converted into amorphous form. Both FTIR spectra and XRD pattern strongly supports the data obtained in EE and drug release studies. Morphology of developed NPSs were analyzed by Scanning electron microscopy. The particles were spherical and smooth in both nanoparticles, however, coated PLGA nanoparticles was bigger in size as compared to plain NPs.

A comparative bioavailability and pharmacokinetic studies were carried out to analyze ELUX contents in rat plasma after oral application of optimized F3, F3C and ELUX suspension. As evident from **Table 2**, dramatic increase (6.8-fold for F3 and 18.5-fold for F3C) in bioavailability is observed in both formulations as compared to normal suspension. Apart from poor solubility, the low bioavailability of ELUX is also due to its low GI permeability (F_GI_ of 2.3%) and first pass effects which produced mainly due to its OATP1B1-mediated uptake (Davenport et al., 2015; Pharmaceutical Industries Ltd., 2015). High increase in bioavailability (*C*_max_, AUC) of F3 and F3C formulation may be due to maximum delayed release of drug bypassing the gastric fluids. Bioavailability enhancement (both rate and extent) was more prominent in F3C formulation and the result was correlated with *in vitro study*, where its high percentage release (86.4%) was observed. Here it is noted that the *C*_max_ and AUC of both F3 and F3C formulation were significantly increased without significant changes in elimination half-life (*T*½). These results suggest that the increase in the relative bioavailability was mainly attributed to the decrease absorption in the intestinal tract and reduce first-pass metabolism in the small intestine and/or liver rather than reduced renal and/or hepatic elimination of ELUX.

## Conclusion

To enhance dissolution rate, bioavailability and maximize the ELUX concentration inside the intestine, initially various plain PLGA NPs were developed and characterized in the current study. Based on average particle size (273.76 nm), PDI (0.327), stable ZP (-30.63 mV) and maximum %EE (56.29%), plain PLGA NPs F3 was optimized and further selected for the preparation coated nanoparticles using enteric Eudragit S100 polymer. The developed coated nanoparticles (F3C) are subjected to *in vitro* release and pharmacokinetic studies in male wistar rats and compared with plain nanoparticles and pure drug. *In vitro* release and pharmacokinetic evaluation in rats showed that F3C sustained the absorption of ELUX compared with plain nanoparticles and pure drug. The oral bioavailability of ELUX from F3C was around 6.8- and 18.5-fold higher than with plain nanoparticles and normal suspension, respectively. These results revealed that the developed coated nanoparticles could be used for its oral delivery for an effective treatment of Irritable Bowel Syndrome with Diarrhea.

## Author Contributions

MA and RA-S setup and carried out formulation development experiments. EE execute statistical analysis. SA and AA performed dissolution experiment and HPLC analysis. MI performed pharmacokinetic study and UPLC-MS/MS analysis.

## Conflict of Interest Statement

The authors declare that the research was conducted in the absence of any commercial or financial relationships that could be construed as a potential conflict of interest.
